# Prognostic significance of AP-2α/γ targets as cancer therapeutics

**DOI:** 10.1038/s41598-022-09494-1

**Published:** 2022-03-31

**Authors:** Damian Kołat, Żaneta Kałuzińska, Andrzej K. Bednarek, Elżbieta Płuciennik

**Affiliations:** Department of Molecular Carcinogenesis, Medical University of Lodz, 90-752 Lodz, Poland

**Keywords:** Cancer, Computational biology and bioinformatics, Genetics, Oncology

## Abstract

Identifying genes with prognostic importance could improve cancer treatment. An increasing number of reports suggest the existence of successful strategies based on seemingly “untargetable” transcription factors. In addition to embryogenesis, AP-2 transcription factors are known to play crucial roles in cancer development. Members of this family can be used as prognostic factors in oncological patients, and AP-2α/γ transcription factors were previously investigated in our pan-cancer comparative study using their target genes. The present study investigates tumors that were previously found similar with an emphasis on the possible role of AP-2 factors in specific cancer types. The RData workspace was loaded back to R environment and 3D trajectories were built via Monocle3. The genes that met the requirement of specificity were listed using top_markers(), separately for mutual and unique targets. Furthermore, the candidate genes had to meet the following requirements: correlation with AP-2 factor (through Correlation AnalyzeR) and validated prognostic importance (using GEPIA2 and subsequently KM-plotter or LOGpc). Eventually, the ROC analysis was applied to confirm their predictive value; co-dependence of expression was visualized via BoxPlotR. Some similar tumors were differentiated by AP-2α/γ targets with prognostic value. Requirements were met by only fifteen genes (*EMX2, COL7A1, GRIA1, KRT1, KRT14, SLC12A5, SEZ6L, PTPRN, SCG5, DPP6, NTSR1, ARX, COL4A3, PPEF1* and *TMEM59L)*; of these, the last four were excluded based on ROC curves. All the above genes were confronted with the literature, with an emphasis on the possible role played by AP-2 factors in specific cancers. Following ROC analysis, the genes were verified using immunohistochemistry data and progression-related signatures. Staining differences were observed, as well as co-dependence on the expression of e.g. *CTNNB1*, *ERBB2*, *KRAS*, *SMAD4*, *EGFR* or *MKI67*. In conclusion, prognostic value of targets suggested AP-2α/γ as candidates for novel cancer treatment. It was also revealed that AP-2 targets are related to tumor progression and that some mutual target genes could be inversely regulated.

## Introduction

Activating enhancer-binding Protein 2 (AP-2) is a family of transcription factors (TFs) belonging to the basic Helix-Span-Helix class (bHSH) of Superclass 1^1^. In homeostatic conditions, its members regulate embryogenesis by managing proliferation, apoptosis or the cell cycle, thus ensuring the correct development of limbs, eyes or facial features^2,3^. However, their altered functionality plays a crucial role in cancer and can be prognostic in oncological patients^4–6^. The fact that each AP-2 member has a different profile within a given tumor, makes this complex field worth investigation. Moreover, an increasing number of reports indicate that there are successful strategies for seemingly “untargetable” transcription factors^7,8^, which opens avenues for the future. Our previous investigations examined the properties of the two best-described AP-2 factors (AP-2α and AP-2γ) in various cancers^9^, they then compared the AP-2α/γ targets between more than twenty tumor types from The Cancer Genome Atlas (TCGA)^10^. The findings shed light on the ability of AP-2α and AP-2γ to regulate the processes underlying the hallmarks of cancer, and the difference between tumor tissues regarding their target genes expression. They also indicated the potential for identifying cancer in cases where normal tissue samples had a distinct expression pattern compared to a corresponding tumor type. The current study provides a further examination of cancer types that were previously found to be similar. The study identifies mutual target genes that might be differently regulated by AP-2α and AP-2γ in specific cancers. It also explores unique target genes (i.e. for each AP-2 factor) that might have prognostic value in these tumors. In addition, it discusses the relevance of AP-2α/γ as candidate transcription factors suitable for cancer treatment.

## Results

### 3D trajectories revealed the dissimilarities across tumor types

**Figure 1:**
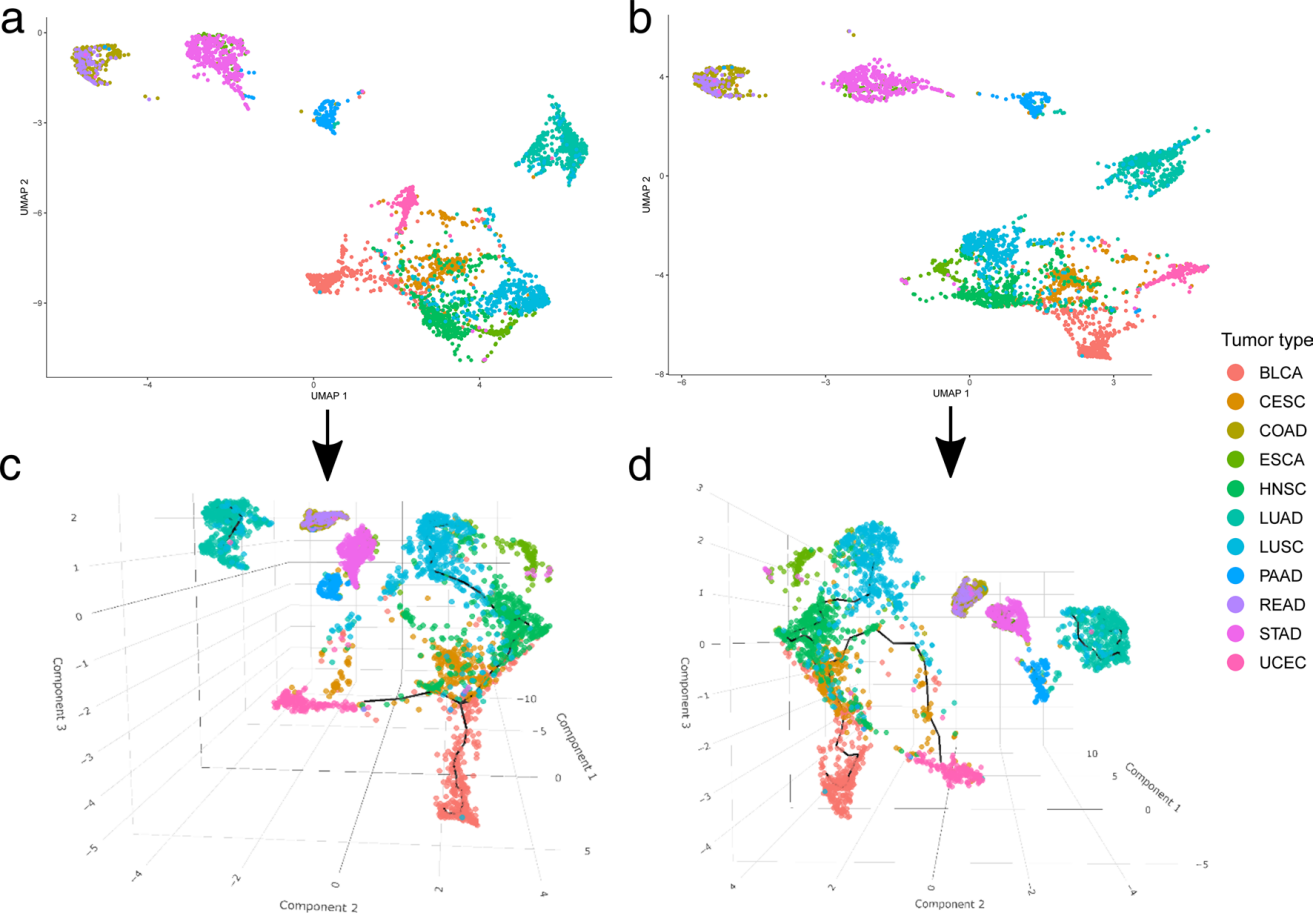
Spatial analysis showing differences between tumors. (**a**) AP-2α target genes list. (**b**) AP-2γ target genes list. (**c**) 3D trajectory of the first subfigure. (**d**) 3D trajectory of the second subfigure. Figure created using Monocle3 (https://cole-trapnell-lab.github.io/monocle3/).

As a preliminary remark, in our previous study^10^ the analysis included global profiling of twenty-one tumors via Monocle3 and was directed to functionally annotate whole gene sets. In the current research, it was decided to focus more specificially on individual AP-2 targets and their significance as cancer therapeutics. Only the tumors that formerly could not be clearly distinguished were included in this study—this has resulted in an analysis which uses RData from a previous study where visualization with Uniform Manifold Approximation and Projection (UMAP) was preliminarily performed. Due to the reduction in the number of cohorts to eleven, the graphs were re-learned to ensure the best dimensional distribution. The distribution was visualized with regard to the full list of gene targets for a given transcription factor; the trajectories are presented in Fig. 1A and B, while the example three-dimensional extensions are visualized in Fig. 1C and D. Complete and interactive 3D plots corresponding to Fig. 1C and D are deposited as Supplementary File S1 and Supplementary File S2, respectively.

**Figure 2:**
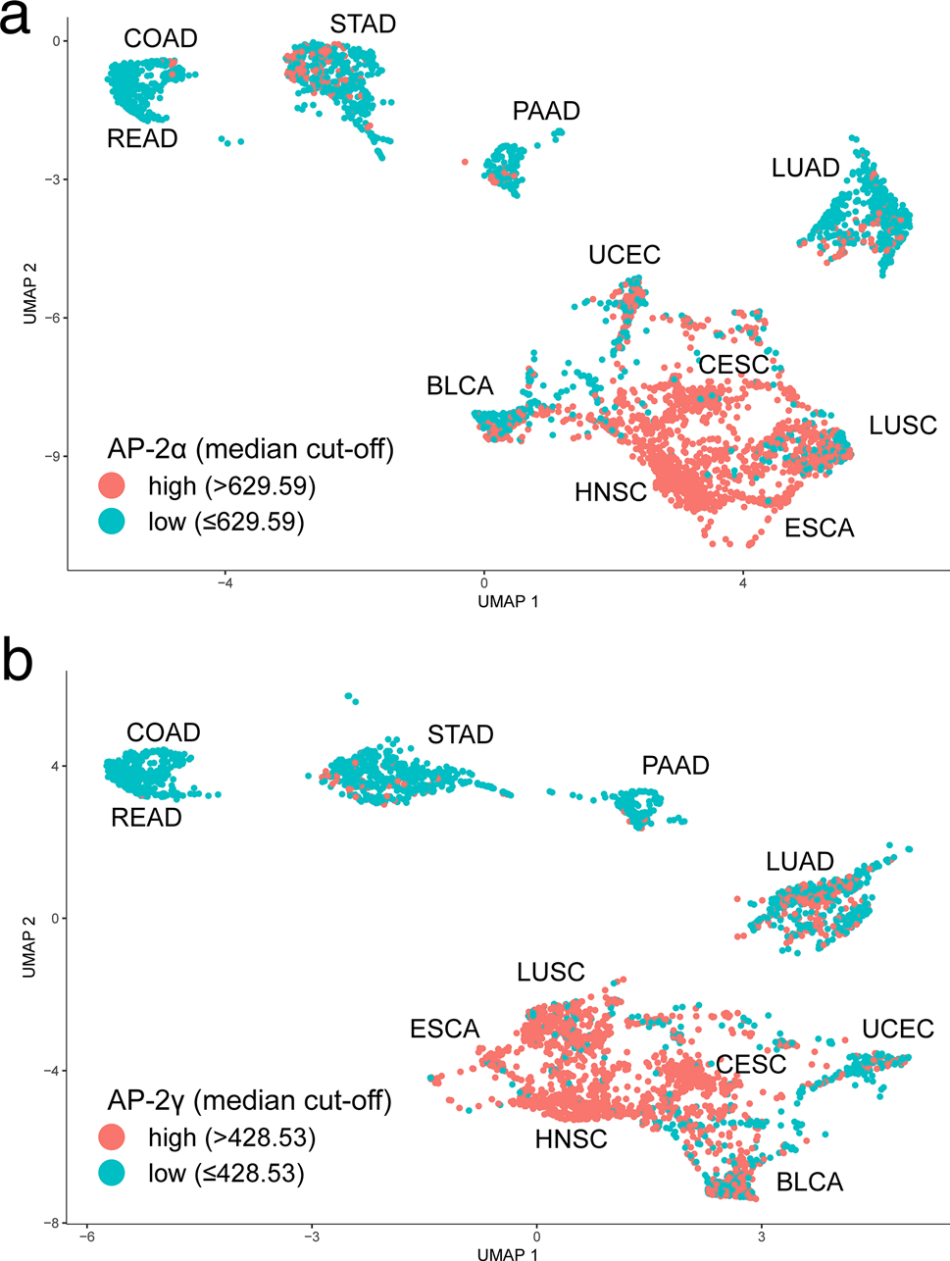
Spatial analysis showing differences between AP-2 factor level. (**a**) AP-2α. (**b**) AP-2γ. Figure created using Monocle3 (https://cole-trapnell-lab.github.io/monocle3/).

Despite being very similar at first glance, subtle differences were observed. For example, some STAD samples were better separated from the rest of the cohort when visualized through AP-2γ target genes. Likewise, UCEC was more distinct from other surrounding cohorts when AP-2α target genes were applied. Nevertheless, the dissimilarities between tumor types can be seen more clearly in 3D trajectories. Additionally, the tumors were grouped based on the expression level of each AP-2 factor (a common median cut-off was applied for all cancers at once). The results indicate that some tumor types demonstrate higher AP-2α/γ expression than others, and that some are more or less uniform in expression (Fig. 2).

For example in STAD or UCEC, a greater number of AP-2α “high” samples can be seen compared to AP-2γ “high”, while in BLCA or LUAD, more AP-2γ “high” samples are found compared to AP-2α. Generally, it seems that both AP-2 factors demonstrate higher expression in the heterogenous mixed cluster (containing BLCA, CESC, ESCA, HNSC, LUSC, UCEC) compared to the other distinct clusters/cohorts.

### Between tumors there are unique target genes of prognostic importance

**Figure 3:**
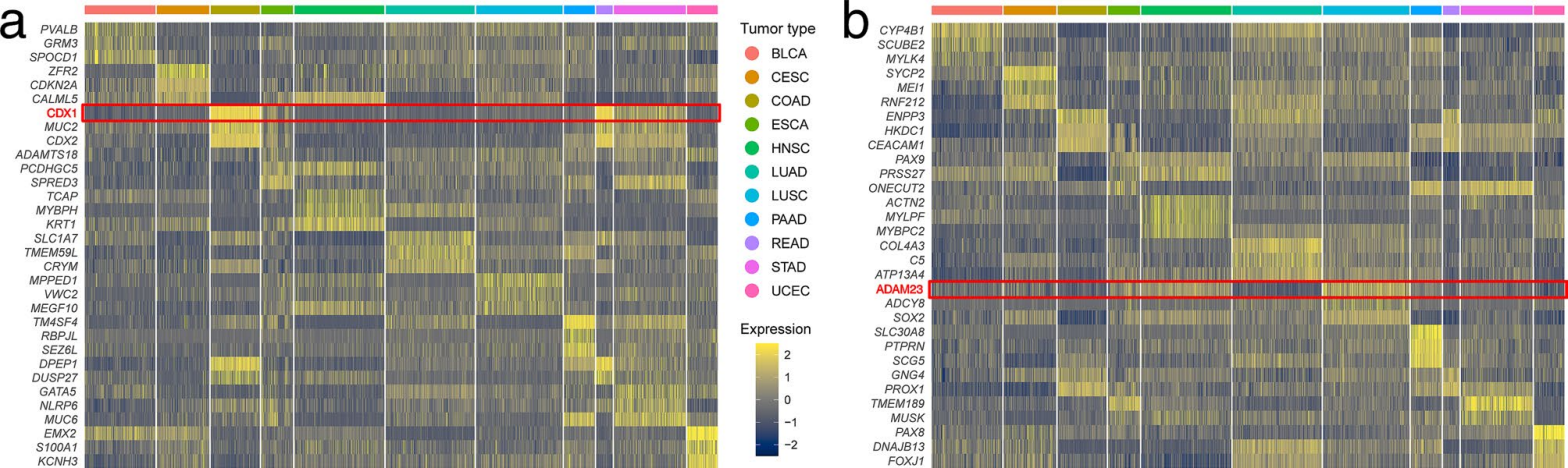
Top three the most specific genes per tumor. (**a**) AP-2α unique targets. (**b**) AP-2γ unique targets. In-text examples are marked with red. Figure created using Seurat 4.0.4 (https://CRAN.R-project.org/package=Seurat).

The study identified unique AP-2α/γ targets between tumors or in a specific cancer. For tumors, this indicated AP-2 target genes which are the most specific for each tumor when compared to others. Only the genes that simultaneously satisfy the demands of specificity, correlation with an AP-2 factor and survival prediction (with external validation) are shown in detail (see penultimate subsection of Results for total number of genes included from each methodology branch). As indicated in Fig. 3A and B (respectively for AP-2α and AP-2γ), our findings indicate that while single gene can be expressed in a few tumors, most are associated more closely with a specific cancer type. For example, *CDX1* (an AP-2α target) is highly expressed in colorectal carcinoma (COAD and READ cohorts) but it is also expressed to a certain extent in STAD. Likewise, the AP-2γ target *ADAM23* is mainly expressed in LUSC but also in ESCA or HNSC. As expected, some tumors demonstrate more specific expression of AP-2 target genes than others.

**Figure 4:**
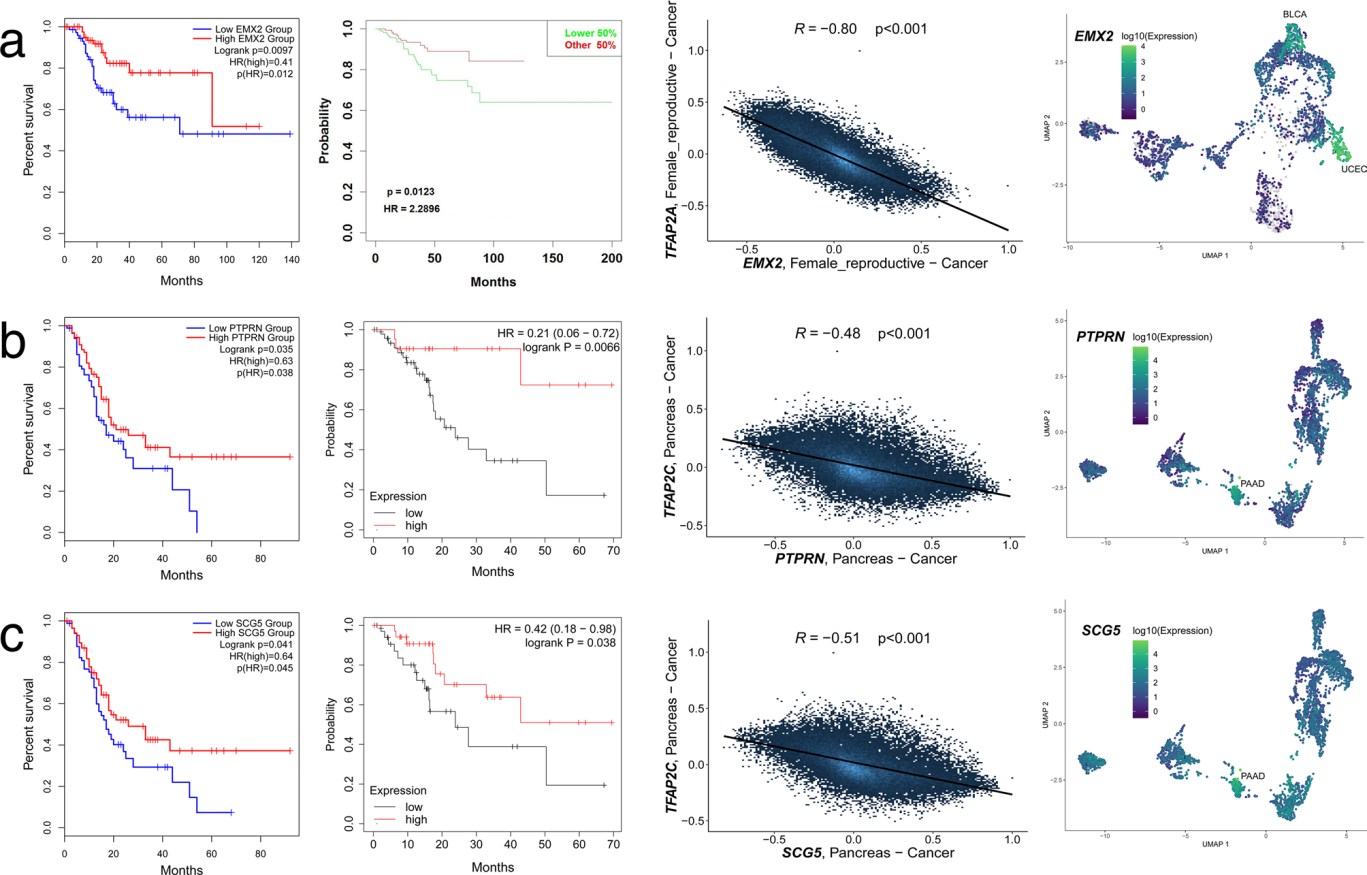
The best three candidate genes from unique targets. (**a**) *EMX2* (AP-2α target). (**b**) *PTPRN* (AP-2γ target). (**c**) *SCG5* (AP-2γ target). Each subfigure contains results from survival analysis (left), which was further validated (middle-left). The candidate gene was correlated with AP-2 factor (middle-right) and the specificity of gene expression in tumors was assessed (right).

All sixty-six genes were subjected to downstream investigation. Three met all the requirements: the AP-2α-regulated *EMX2* for UCEC, and the AP-2γ targets *PTPRN* and *SCG5* for PAAD. Not only were they negatively correlated with the corresponding AP-2 factor, but they also significantly affected Disease-Free Survival (DFS), as confirmed externally. Regarding specificity, *PTPRN* was found to be the most specific (0.73), followed by *EMX2* (0.72) and *SCG5* (0.65); this can be seen in Fig. 4 (the last graph in each subfigure).

### Unique targets can predict patient survival for a specific cancer type

Unique AP-2α or AP-2γ targets were also independently analyzed within a specific cancer by classifying AP-2α/γ expression as “high” or “low”, with regard to a median cut-off point. The three genes with the highest specificity per “high” or “low” group were identified for each AP-2 factor, giving six genes per tumor. As some AP-2 targets did not meet the minimal specificity requirement (Table 1), fewer genes were included in the downstream analysis.

**Table 1:** The top three most specific genes per “high” or “low” group in tumors for both AP-2α and AP-2γ.

Transcription factor	Tumor	“High” group	“Low” group
**AP-2α**	BLCA	***KRT1****, ****KRT14****, ****NOTUM***	***TCAP****, ****SLC16A12****, ****EMX2***
	CESC	*KRT14, GLDC, SOX15*	***DPEP1****, ****TTLL10****, ****NOTUM***
	COAD	***MUC6****, ****SLC26A9****, ****TM4SF4***	***TCAP****, ****TMEM132C****, ****ISM2***
	ESCA	***KRT1****, ****KRT14****, ****CALML5***	***NLRP6****, ****DPEP1****, ****CDX1***
	HNSC	***KRT1****, ****PCDHGC5****, ****PLA2G4D***	***MUC6****, ****NOTUM****, ****ALDH1A1***
	LUAD	***KRT14****, ****COL7A1****, ****LAMA1***	***NOTUM****, ****KCNE4****, ****SLC16A12***
	LUSC	*PCDHGC5, VASH2, KRT14*	***NOTUM****, ****DLL3****, ****TMEM59L***
	PAAD	***MUC2****, ****KRT14****, CGB5*	***SLC12A5****, ****GRIA1****, ****SEZ6L***
	READ	***GRIK2****, ****TM4SF4****, SHC2*	*EDA, GPR55, SCN4A*
	STAD	***TGM1****, ****S100A2****, ****KRT14***	***SMTNL2****, ****SLC16A12****, ****PYGM***
	UCEC	***KRT14****, ****ADAMTS18****, ****KCNH3***	***DLL3****, SYN1, ADAM33*
**AP-2γ**	BLCA	*MYLK4, ALOXE3, CEACAM6*	***FAM78B****, SIGLEC15, PAX8*
	CESC	***PDE6A****, ****PRIMA1****, AKR1B15*	***HKDC1****, ****HABP2****, ****PAX8***
	COAD	***FAM83A****, PTPRN, PRIMA1*	*KCNK17, ENPP3, STAG3*
	ESCA	***AKR1B15****, ****CDH26****, ****ALDH3B2***	***HABP2****, ****NPC1L1****, ****FOXA3***
	HNSC	***GSTM5****, ****GSDMA****, PLA2G4E*	***INSM1****, ****SLC7A2****, ****KCNJ10***
	LUAD	*KRT15, PRSS27, SCUBE3*	***NPC1L1****, ****INSM1****, ****PPEF1***
	LUSC	*PRSS27, AKR1B15, SYCP2*	***INSM1****, ****SPIB****, ****ENPP3***
	PAAD	*FAM83A, IRX3, PTGES*	***COL11A2****, ****ARX****, ****VGF***
	READ	***CYP24A1****, ****MYO3A****, FPR1*	***F5****, RPL39L, HKDC1*
	STAD	***PRSS27****, ****KRT15****, ****FAM83A***	***SPIB****, CCR7, KCNJ10*
	UCEC	*VIPR1, RUNDC3B, COL14A1*	***ONECUT2****, ****CEACAM6****, ****COL4A3***

Underlined and in bold are genes that met > 0.6 requirement in terms of specificity estimated by top_markers().

**Figure 5:**
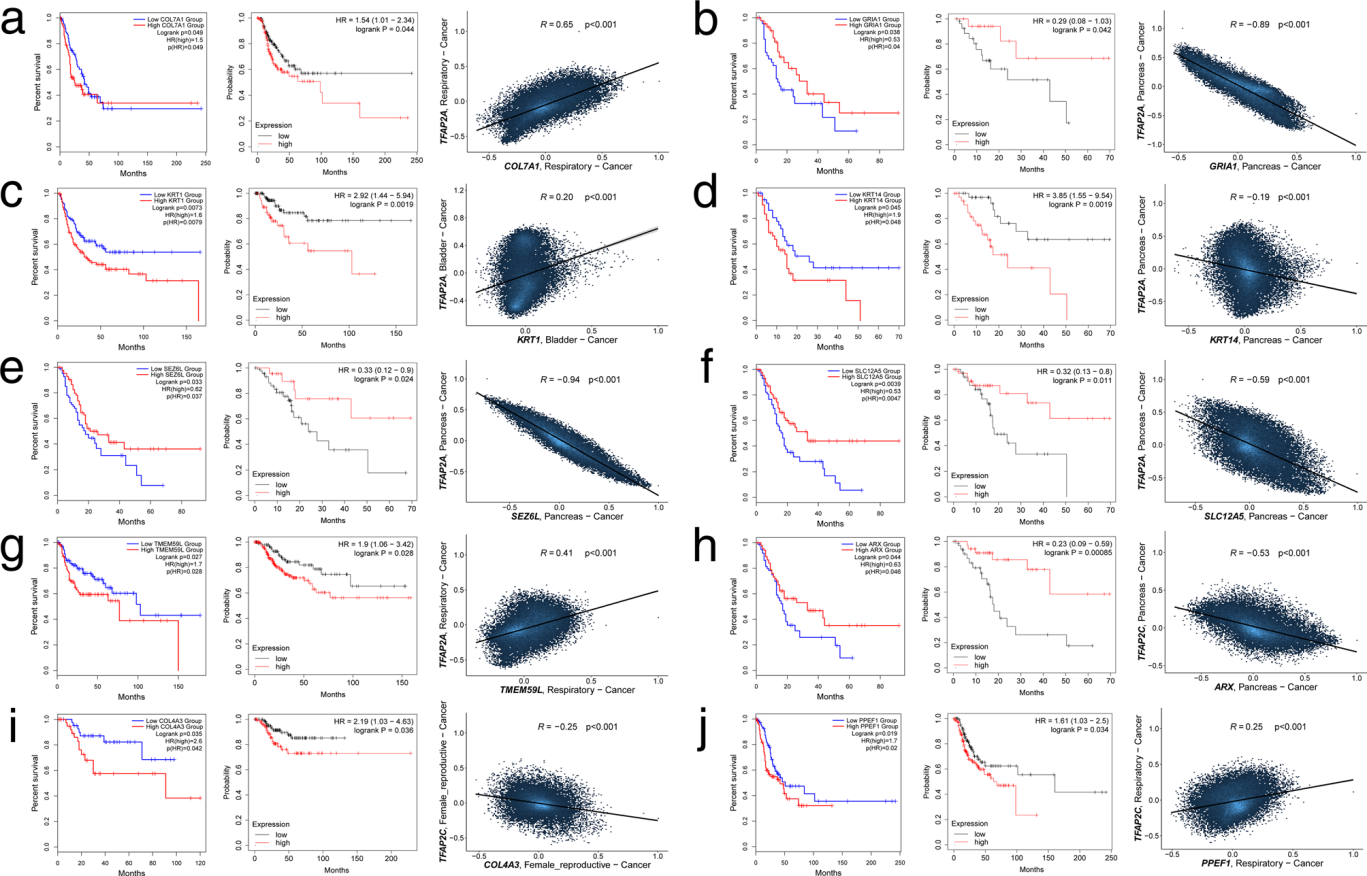
Relevant genes identified within a specific cancer. (**a**) *COL7A1* (AP-2α target). (**b**) *GRIA1* (AP-2α target). (**c**) *KRT1* (AP-2α target). (**d**) *KRT14* (AP-2α target). (**e**) *SEZ6L* (AP-2α target). (**f**) *SLC12A5* (AP-2α target). (**g**) *TMEM59L* (AP-2α target). (**h**) *ARX* (AP-2γ target). (**i**) *COL4A3* (AP-2γ target). (**j**) *PPEF1* (AP-2γ target).

Eventually, it was found that seven targets of AP-2α (*KRT1, COL7A1, TMEM59L, KRT14, SLC12A5, GRIA1, SEZ6L*) were of prognostic importance for patients having BLCA, LUAD, LUSC or PAAD. Regarding genes regulated by AP-2γ, three targets (*PPEF1, ARX, COL4A3*) had prognostic value for individuals from LUAD, PAAD or UCEC cohort. Most of the AP-2α unique targets concerned PAAD; interestingly, they were all negatively correlated with transcription factor-encoding gene. In contrast, the remaining part (*KRT1* for BLCA, *COL7A1* for LUAD and *TMEM59L* for LUSC) was positively correlated with AP-2α. When it comes to AP-2γ, it correlated positively with *PPEF1* and *ARX* while negatively with *COL4A3*. Figure 5 presents a summary for targets of both TFs.

### Differently-regulated mutual target genes were found in ESCA and LUAD

**Figure 6:**
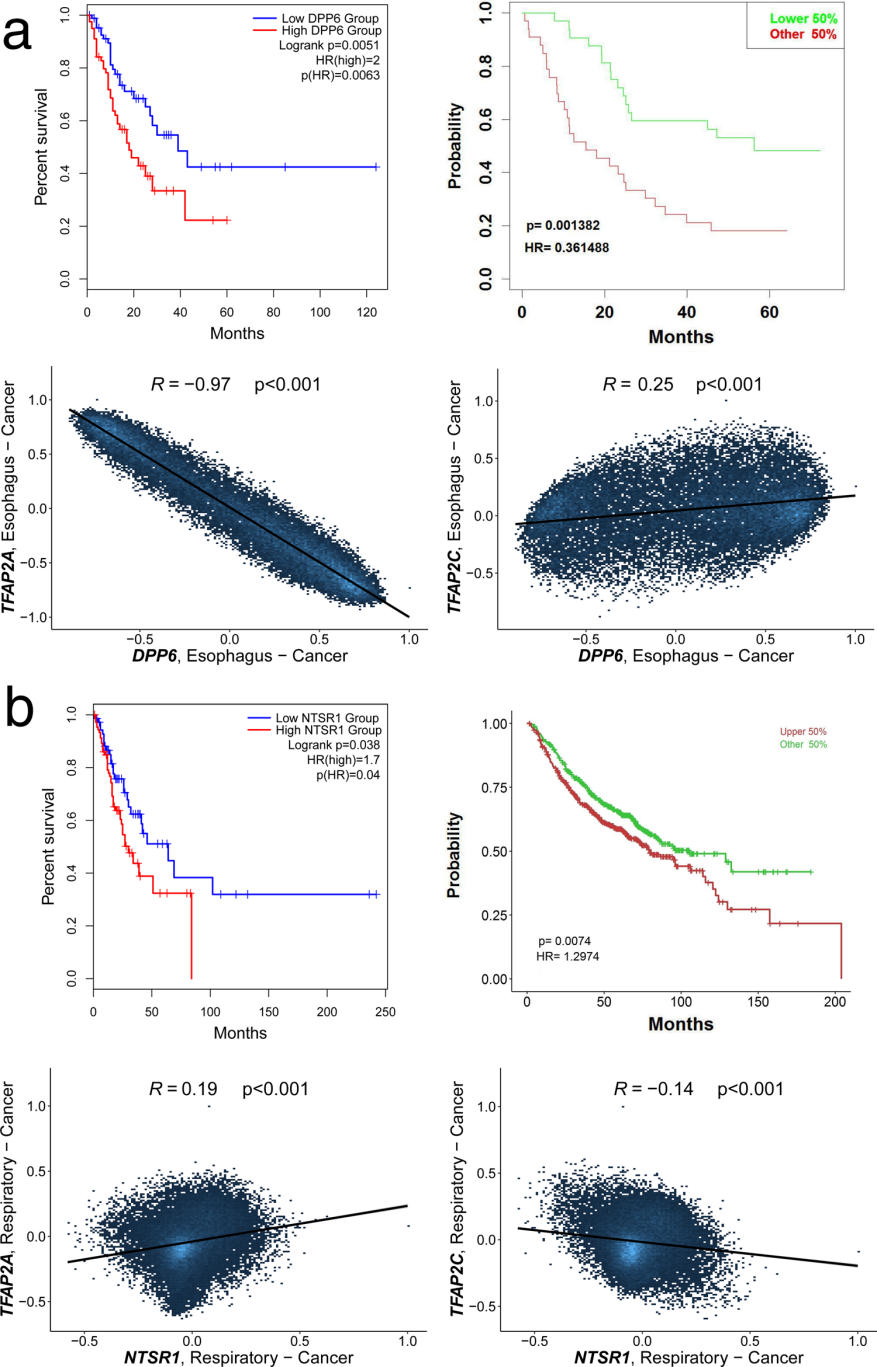
Differently regulated mutual targets. (**a**) *DPP6*. (**b**) *NTSR1*.

To identify genes whose expression could be regulated in opposite directions by AP-2α and AP-2γ within a single tumor type, each cancer was examined individually. Samples of specific tumor were compared regarding high/low phenotypes for the two AP-2 factors; this allowed changes in expression of mutual target genes to be established between phenotypes. Only two genes satisfied all criteria: *DPP6* and *NTSR1*. AP-2α strongly downregulates the former (Fig. 6A), while AP-2γ appears to downregulate the latter (Fig. 6B). High *DPP6* expression is associated with shorter survival in ESCA patients and *NTSR1* with shorter survival in LUAD.

### ROC curves confirmed that most of the selected genes are good predictors

**Figure 7:**
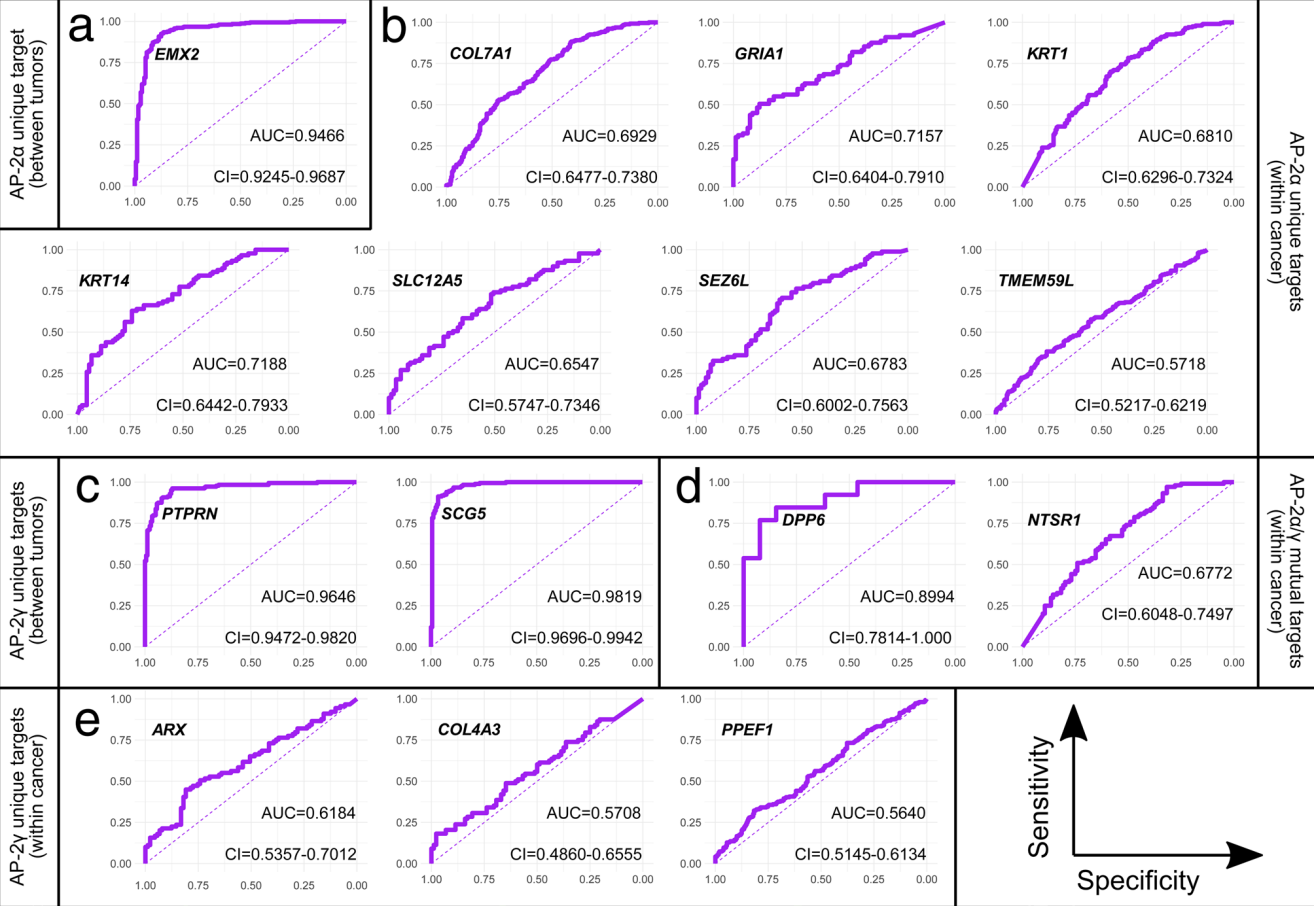
Receiver operating characteristic curves of genes that met the study requirements. (**a**) Unique for AP-2α (between-tumor comparison). (**b**) Unique for AP-2α (comparison within a specific cancer type). (**c**) Unique for AP-2γ (between-tumor comparison). (**d**) Mutual for AP-2α and AP-2γ. (**e**) Unique for AP-2γ (comparison within a specific cancer type).

The relevant targets from previous sections were first demonstrated in Table 2 to infer the possible AP-2 role in specific cancer, and then were subjected to Receiver Operating Characteristic (ROC) analysis to confirm predictive abilities. Binary classification depended on methodology branch e.g. for genes identified within a specific cancer the “high” and “low” groups of AP-2 factor(s) were used. The targets identified by the tumor vs tumor comparisons (*EMX2, PTPRN, SCG5*) required a representative cohort for ROC analysis; these three genes concern only UCEC and PAAD tumors. For *EMX2* expression, UCEC was compared with CESC, an other carcinoma of the female reproductive system. For *PTPRN* and *SCG5* expression, PAAD was compared to ESCA, since it was the closest cohort in terms of size (no tumor with a similar site of origin to PAAD was included in this study). Out of fifteen genes that were suitable for ROC analysis, *COL4A3*, *PPEF1* and *TMEM59L* did not predict a binary outcome sufficiently: their Area Under the Curve (AUC) was respectively 0.57, 0.56 and 0.57. The usefulness of *ARX* is also questionable since the AUC was 0.61. Nevertheless, the remaining AP-2 targets presented AUC > 0.65 including some even above 0.9. ROC curves are collected in Fig. 7.

**Table 2:** Supposed role of AP-2α and AP-2γ in cancer based on genes that met the study requirements.

Transcription factor	Tumor	Gene of interest	Prognosis when gene is highly expressed	Correlation with AP-2 factor	Possible role of AP-2 factor in cancer
**AP-2α**	BLCA	*KRT1*	Unfavorable	Positive	Pro-tumorigenic
	ESCA	*DPP6*	Unfavorable	Negative	Anti-cancer
	LUAD	*COL7A1*	Unfavorable	Positive	Pro-tumorigenic
		*NTSR1*	Unfavorable	Positive	Pro-tumorigenic
	LUSC	*TMEM59L*	Unfavorable	Positive	Pro-tumorigenic
	PAAD	*KRT14*	Unfavorable	Negative	Anti-cancer
		*GRIA1*	Favorable	Negative	Pro-tumorigenic
		*SEZ6L*	Favorable	Negative	Pro-tumorigenic
		*SLC12A5*	Favorable	Negative	Pro-tumorigenic
	UCEC	*EMX2*	Favorable	Negative	Pro-tumorigenic
**AP-2γ**	ESCA	*DPP6*	Unfavorable	Positive	Pro-tumorigenic
	LUAD	*NTSR1*	Unfavorable	Negative	Anti-cancer
		*PPEF1*	Unfavorable	Positive	Pro-tumorigenic
	PAAD	*ARX*	Favorable	Negative	Pro-tumorigenic
		*PTPRN*	Favorable	Negative	Pro-tumorigenic
		*SCG5*	Favorable	Negative	Pro-tumorigenic
	UCEC	*COL4A3*	Unfavorable	Negative	Anti-cancer

### Immunohistochemistry showed staining differences and the genes were found to be related to tumor progression

**Figure 8:**
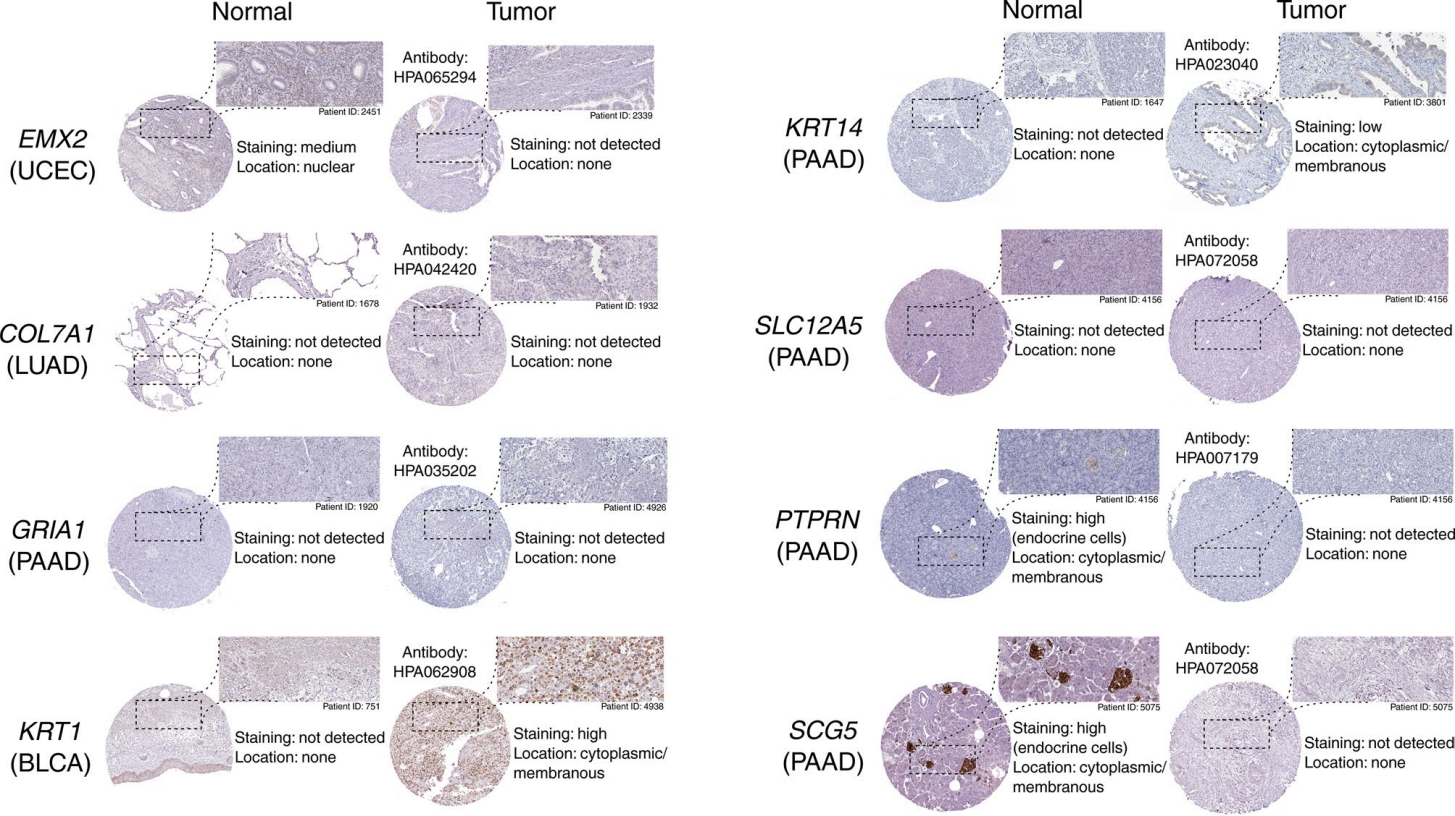
Representative immunostaining data for post-ROC genes from both normal and tumor tissues. For each gene, the cohort abbreviation for which it was identified throughout the study is given in brackets.

To complement the results of the survival analysis, all genes that met the AUC requirement (herein denoted as post-ROC genes) were subjected to further analysis of their immunohistochemical (IHC) data (Fig. 8). Out of eleven genes, there was insufficient or no data for *DPP6* or *SEZ6L* and *NTSR1* in the tumor tissues for which they were identified throughout the study; thus, no comparison was possible. For the remainder, three genes (*SLC12A5, COL7A1, GRIA1*) showed no differences between tumor and normal tissue, while five of them (*EMX2, KRT1, KRT14, PTPRN, SCG5*) presented various staining.

**Figure 9:**
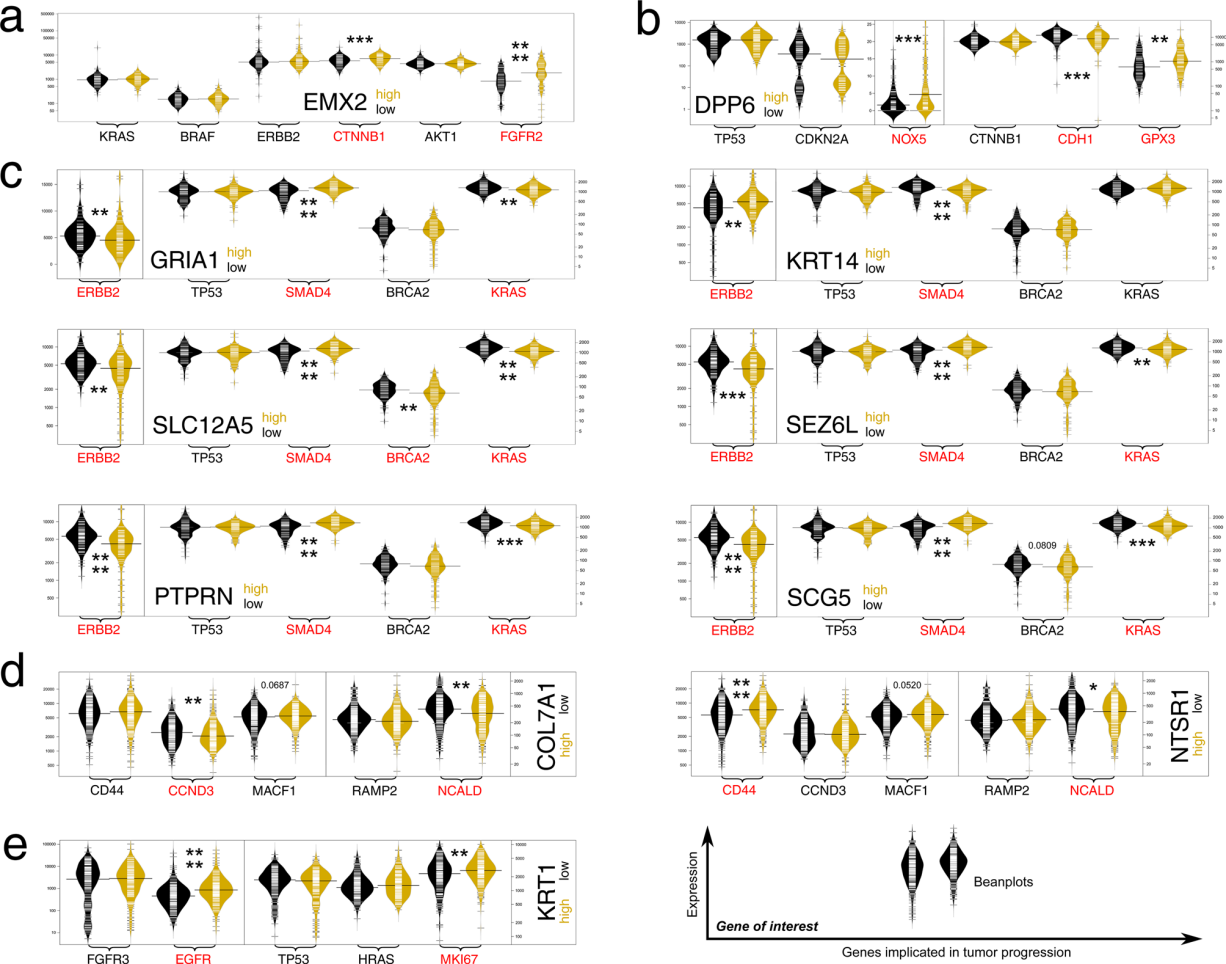
Co-dependence of post-ROC genes and tumor progression-related genes. Genes with an impact on patient survival in: (**a**) UCEC, (**b**) ESCA, (**c**) PAAD, (**d**) LUAD, (**e**) BLCA. In most cases, two separate scales (separated by a continuous line) are used on the Y axis due to large differences in expression level.

The study also examined whether the expression of the post-ROC genes could co-depend on expression of other genes known to play a role in tumor progression (a group of five or six genes was selected for each tumor based on literature data). It was found that patients with high or low expression of each post-ROC gene presented varied expression of at least two progression-related genes (Fig. 9).

## Discussion

Apart from their role in human development, AP-2 transcription factors are also known to influence carcinogenesis; as such, they have prognostic value for cancer patients^11,12^. Once regarded as “undruggable” DNA-binding proteins, transcription factors are now the object of studies examining selective modulators of their activity^8,13,14^. Among these factors, AP-2 is of particular significance since one of the members from this family, AP-2δ (encoded by the *TFAP2D* gene), is one of less than three hundred candidate transcription factors suitable for studies focused on examining selective modulators of transcription factor activity^15^.

This suggests that other AP-2 representatives also deserve attention with regard to TF-based targeted therapy. Our previous research on AP-2α and AP-2γ in twenty-one tumors from TCGA identified evident differences in the expression of their target genes between tissues^10^. The present study examined tumors whose independence was not so obvious. The identification of AP-2α/γ mutual or unique targets provided an indication of their relevance for a specific cancer and indirectly, whether AP-2α or AP-2γ might be worth consideration in forthcoming TF-based therapy.

To identify the most relevant AP-2α/γ targets, mutual and unique genes were considered separately. Only fifteen genes met the initial requirements of this study; some were excluded based on ROC curves. Prior to ROC analysis, these fifteen genes were confronted with the literature data, revealing their potential in cancer therapy. Below, they are discussed in sequence.

One of methodology branches was to investigate unique AP-2α/γ target genes between tumors. Only *EMX2* (for AP-2α) and *PTPRN* or *SCG5* (both for AP-2γ) were identified; however, they demonstrated very good discriminatory properties. *EMX2* encodes a homeodomain-containing transcription factor essential for growth and differentiation^16^. It is also a fundamental protein necessary for the development of the reproductive tract^17,18^; it is therefore not surprising that it was found to be specific for uterine corpus endometrial carcinoma in the current study: *EMX2* expression was noted mainly within UCEC and to a small extent within BLCA. High expression was found to be favorable for survival in UCEC patients, which is in agreement with previous data indicating that it acts as a tumor suppressor in lung, kidney, colorectal, gastric cancers or in sarcoma^16,19–22^. Moving forward, *PTPRN* encodes transmembrane receptor-type protein tyrosine phosphatase, mainly expressed in neuroendocrine tissues such as pancreas^23^. Its expression was found to be PAAD-specific in this study. High *PTPRN* expression was found to be favorable for survival in pancreatic adenocarcinoma patients. This contradicts data from other tumors; for example, its presence favors metastasis and migration promotion in LUAD^24^, and it is associated with worse prognosis of patients with hepatocellular carcinoma^25^ or progression of gastric cancer^26^. However, little or no research has been performed on the role of *PTPRN* in PAAD, and it may be the case that the context is tissue-dependent, similar to *GLDC* gene function across tumors^27^. Lastly, *SCG5* gene encodes secretogranin V, an essential chaperone involved in signaling that influences proliferation, among other things^28,29^. *SCG5* was found to be implicated in polyposis syndrome, which is associated with colorectal cancer (CRC)^30^. Our present findings indicate that *SCG5* expression was specific for the PAAD cohort. This is in line with data regarding ability of secretogranin V to regulate prohormone convertase 2 (PC2), a neuroendocrine-specific proteinase^31^. In addition, the higher *SCG5* expression was found to improve survival among PAAD patients, which is consistent with the literature^32^.

The other group of unique AP-2α/γ targets identified throughout this study were derived from comparisons within a specific cancer. From genetic information of *COL7A1*, the alpha chain of basement-membrane protein, type VII collagen, is formed. Its high expression was found to be unfavorable for survival among lung adenocarcinoma patients. Despite the lack of literature data on the role of *COL7A1* in LUAD, similar observations have been made in patients with squamous-cell skin cancer^33^, laryngeal cancer^34^ and gastric cancer^35^. In contrast, high expression of *GRIA1*, an ionotropic receptor involved in glutamate signaling, was found to be beneficial in PAAD; indeed, *GRIA1* has previously been included in a gene-based risk score system constructed for patients with that tumor^36,37^. However, its function may depend on tissue type, since *GRIA1* was shown to promote tumor progression in glioma^38^. Moving forward, *KRT1* encodes a member of the intermediate filament superfamily, i.e. the clade comprising the cytokeratins: common markers of differentiation, migration and proliferation in epithelial cells^39^. *KRT1* is used to molecularly distinguish muscle-invasive urothelial carcinoma into luminal or basal subtypes^40^; as such, the identification of this target as an unfavorable prognostic marker in BLCA is useful. *KRT1* was previously found to be involved in bladder tumorigenesis^41^, and to be associated with advanced tumor stage and worse prognosis in melanoma patients^42^. The other cytokeratin, *KRT14*, was also found to worsen the outcome in patients with PAAD. This is in line with data from ovarian and lung cancers^43,44^; however, a study on pancreatic carcinoma suggests that *KRT14* is not predictive of outcome (hazard ratio was similar as in our study but statistical significance was not met)^45^. Another gene indicated in the PAAD cohort was *SEZ6L*, encoding a transmembrane protein implicated in signal transduction, protein–protein interactions and complement regulation (via inhibition of C3 convertases and promotion of C3b degradation)^46^. High expression was favorable for PAAD patients, which is in line with other studies on pancreatic carcinoma^47^ or lung cancer^48^. Similarly, a potassium chloride cotransporter encoded by *SLC12A5,* also improved DFS in PAAD; however, no previous research exists on this tumor (various members of the solute carrier family have been discussed in pancreas neoplasm but *SLC12A5* is not among them^49^). In fact, previous studies indicate that this gene promotes tumor invasion and metastasis in BLCA^50^ or proliferation and G_1_/S cell cycle transition in colon cancer^51^. The last target unique for AP-2α was the transmembrane protein-encoding gene *TMEM59L* (also known as *C19orf4*). However, its role in LUSC and other cancers remains unclear^52^. The protein is believed to regulate apoptosis^53^ and the GeneCards website indicates that *TMEM59L* modulates the glycosylation of amyloid precursor protein. The remaining genes from unique targets were found to be dependent on AP-2γ. *ARX* encodes a transcription factor that is crucial in regulating the endocrine pancreas development; it is mainly expressed in the central nervous system, skeletal muscles and aforementioned lineage of pancreatic cells^54,55^. This gene correlates with an aggressive course and frequent relapses in pancreatic neuroendocrine tumors^56,57^. Our present survival analysis suggests that high *ARX* expression is favorable for PAAD patients, which might suggest a subtype-specific behavior that is worth investigation since pancreatic tumors arising from endocrine or epithelial portion have different properties^58–60^. The prognostic outcome of *COL4A3* could also vary depending on cancer type. Our findings indicate that high expression was associated with shorter survival of UCEC patients. This corresponds to research on non-small cell lung (NSCLC) and breast cancers^61,62^ but contradicts data from HNSC^63^. Finally, while the molecular function of phosphatase encoded by *PPEF1* is not known in detail, it has been connected to apoptosis regulation and the response to calcium (Ca^2+^ is also second messenger controlling cell death)^64^. It exerts a tumorigenic role in breast cancer^65^ and was found to be overexpressed in pancreatic carcinoma^66^ which confirms both its prognostic value in LUAD (poorer survival when highly expressed) and literature data regarding lung cancer^64^.

The presence of any mutual targets that are differently regulated by AP-2α and AP-2γ within a specific cancer type was also investigated. Only two genes were found to be inversely regulated by both transcription factors and met all the other requirements: *DPP6* (in ESCA cohort) and *NTSR1* (in LUAD). The first gene stores information about membrane glycoprotein of dipeptidyl peptidase IV family which regulates apoptosis, differentiation or proliferation^67,68^. Our present findings indicate that high *DPP6* expression worsens survival of ESCA patients which is in line with previous data regarding colon cancer progression^67^. However, as no DFS data was present in the validation cohort, the survival analysis of DFS with regard to *DPP6* level was validated using overall survival outcome. Interestingly, this gene was found to be overexpressed in long-term survivors of study on esophageal cancer compared to those with shorter survival^69^, and *DPP6* hypomethylation or hypermethylation has been noted depending on cancer type^67^. It is undoubtedly an important gene in esophagus neoplasms since, in addition to opposite regulation by AP-2α/γ, its expression is regulated by ARID3A, ZNF354C: two out of five key transcription factors crucial for carcinogenesis and development of esophageal squamous cell carcinoma^70^. Moreover, the gene encoding AP-2α (*TFAP2A*) was also found to significantly correlate with longer survival rate^70^. Considering how strong *TFAP2A* and *DPP6* correlated in this study (R = − 0.97) and that AP-2γ is suspected to contribute in esophageal cancer progression^9^, they clearly deserve further investigation in ESCA. Last but not least, *NTSR1* gene encodes the seven-transmembrane G-protein coupled receptor, through which neurotensin acts on proliferation, DNA synthesis or migration^71^. This receptor has been the subject of more study than other neurotensin receptors^72^; it has been found to play a tumorigenic role in PAAD, HNSC, NSCLC or CRC^73,74^. In LUAD, it has been found to correlate with poor prognosis and to participate in cancer progression^71,75^; this corresponds to the survival analysis performed in this study. As such, *NTSR1* has often been proposed as a potential therapeutic or diagnostic target^71,74,76^.

All genes with satisfactory AUC were subjected to analysis of IHC data. Their influence on tumor progression was also evaluated; each cancer was considered separately, with progression-related genes being selected for BLCA^77^, ESCA^78^, LUAD^79^, PAAD^80,81^ and UCEC^82^ based on literature. The prognostic value of *EMX2* suggests it as a favorable marker for DFS and the staining confirms that it is present at a higher level in normal endometrial tissue than in tumor, as noted previously^83^. Similarly, high *PTPRN* or *SCG5* expression was found to be favorable for PAAD patients; more intense staining was observed in normal pancreatic tissue, suggesting their expression is lowered during tumorigenesis. In addition, *SCG5* expression has been found to be decreased in primary pancreatic cancer, and even lower in metastatic carcinoma^32^. In contrast, *KRT1* or *KRT14* staining was more intense in BLCA or PAAD than in corresponding normal specimens, confirming their observed unfavorable impact on patient outcome. *KRT1* was found to be elevated in urospheres (which contain cancer initiating cells) compared to the parent non-tumorigenic UROtsa cell line, which was exposed to arsenite in order to acquire transformed cells^84^. Likewise, basal keratins (including *KRT14)* are expressed in a subset of pancreatic ductal adenocarcinoma but are undetectable in normal pancreas^45^.

The observations regarding the co-dependence of post-ROC genes and tumor progression-related genes are generally consistent with both prognostic outcome and IHC data; however, *EMX2* presented intriguing tendencies. Namely, while survival analysis and immunohistochemistry are both consistent with literature data suggesting *EMX2* as endometrial tumor suppressor, the *EMX2*^high^ group of patients were found to have higher expression of *CTNNB1* and *FGFR2*, two known oncogenes implicated in endometrial oncogenesis^82^. This clearly requires future investigation. Nevertheless, the three genes identified in PAAD for which IHC data was available (*PTPRN, SCG5, KRT14*) indicated that an important switch exists between *ERBB2* and *SMAD4* expression i.e. the oncogene and tumor suppressor, respectively^80,81^. If the expression of *PTPRN* and *SCG5* (both favorable for prognosis) was high, *SMAD4* is elevated but *ERBB2* is lowered, similar to the *KRAS* oncogene. In contrast, when *KRT14* expression (being unfavorable) is high, the opposite tendency is seen for *ERBB2* and *SMAD4*. Similar observations were made for the genes *SEZ6L, GRIA1* and *SLC12A5* in PAAD, whose impact on survival suggested anti-cancer properties but insufficient data or no differences were observed in IHC specimens. The same changes in *ERBB2*, *SMAD4* and *KRAS* level are observed between the “high” and “low” groups of these genes and conforms that of *PTPRN* and *SCG5*. The remaining genes in which IHC data was insufficient or staining was not detected, concern *DPP6* (for ESCA) and *COL7A1* or *NTSR1* (both for LUAD), all being unfavorable for patient outcome. High *DPP6* expression was found in the group that also demonstrated increased *NOX5* and *GPX3*; while both genes belong to the same biological pathway i.e. regulate reactive oxygen species levels, the former gene is upregulated during esophageal carcinogenesis while the latter is downregulated^85^. Nevertheless, the metabolism of oxygen by-products is dysregulated and this is known to affect tumor progression^86,87^. Undoubtedly, patients with high *DPP6* expression demonstrate altered adhesion, as *CDH1* expression is significantly decreased, which is associated with poor survival^88^. In LUAD, “high” expressing groups of both *COL7A1* and *NTSR1* demonstrated reduced *NCALD,* the gene whose low expression worsens patient outcome^89^. Furthermore, *CCND3,* whose high expression improves survival^79^, was reduced in the *COL7A1*^high^ group while *CD44*, a promoter of KRAS-dependent lung tumorigenesis^90^, was elevated in the *NTSR1*^high^ group. Lastly, *KRT1*^high^ BLCA patients had significantly higher expression of *EGFR* and *MKI67*, suggesting that proliferation is potentiated in this group; this complements the survival analysis (high *KRT1* is unfavorable in BLCA) and IHC data (*KRT1* staining is higher in cancer than normal specimens).

Finally, there is a need to determine whether AP-2α/γ upregulate or downregulate all the above genes, as estimated by correlation analysis, and thus the role of AP-2 factors in particular cancer types. It appears that while both anti-cancer and pro-tumorigenic roles are possible, the latter is predominant (Table 2), suggesting AP-2α/γ are potential candidates for cancer treatment. Moreover, the presence of “anti-cancer” next to “pro-tumorigenic” within a single tumor only complicates the final conclusion regarding AP-2 role in that cancer (Table 2; e.g. *KRT14* vs *GRIA1, SEZ6L, SLC12A5* for AP-2α within PAAD or *NTSR1* vs *PPEF1* for AP-2γ within LUAD). The only unequivocal “anti-cancer” cases are *DPP6* in ESCA (for AP-2α) or *COL4A3* in UCEC (for AP-2γ) but this cannot be concluded only on the basis of the single TF–target example. However, three genes per AP-2 factor were of favorable prognostic value for PAAD and all were negatively regulated by the TF, implying both AP-2α and AP-2γ could play tumorigenic role in this tumor. Our previous findings suggest that AP-2α may have an oncogenic role in pancreatic cancer^9^; as such, further studies are needed of these two AP-2 factors in this tumor type. In addition, they may have different roles in other neoplasms and further research could confirm their value as novel candidate TFs suitable for targeting in cancer treatment, as aforementioned in terms of AP-2δ^15^.

## Conclusions

Our findings indicate that genetic targets of AP-2α and AP-2γ differ between seemingly similar tumors. These differences can be of prognostic importance, being implicated in tumor progression, and they may be of value in targeted therapy. The study also paved the way for these two AP-2 transcription factors to be considered as candidates for developing TF-based cancer treatment.

## Methods

### Data collection, identification of AP-2 target genes, building trajectories

**Figure 10:**
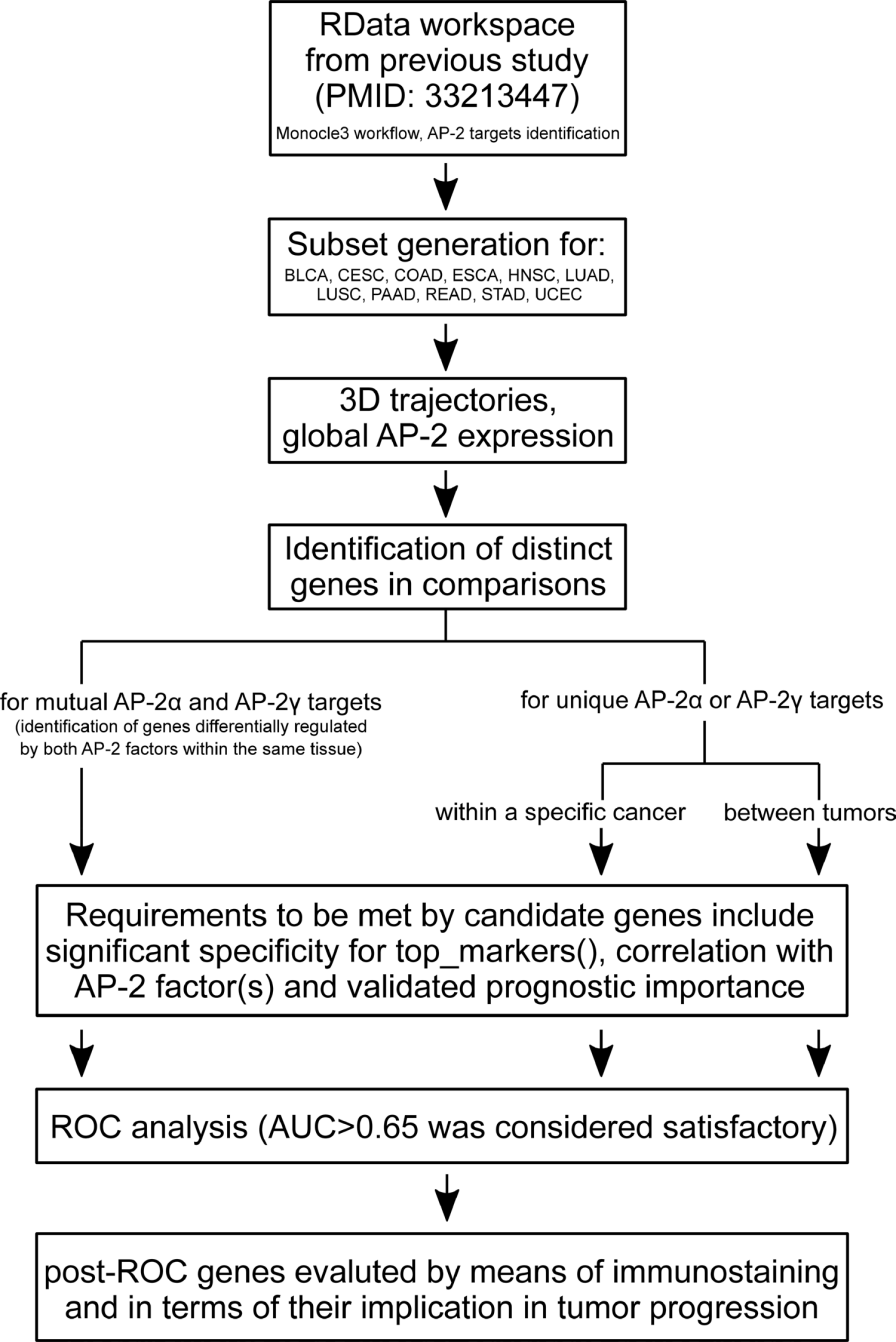
Methodology overview.

The expression and clinical data of patients (level 3 RNA-seqV2, RSEM normalized) from TCGA-dedicated GDAC Firehose Repository (gdac.broadinstitute.org), together with a list of AP-2α/γ targets (combined from GTRD v19.10, TRANSFAC v2019.2 and TRRUST v2—see Supplementary File S3) were loaded back to R environment in the form of RData workspace (available at github.com/koldam/AP2-prognostic-significance). Thus, the entire workflow (with objects) of the Monocle3 R toolkit (cole-trapnell-lab.github.io/monocle3), performed in the former research^10^, was automatically exported. The additional (i.e. not previously performed) part within Monocle3 included e.g. the generation of subsets using choose_cells(). At first, the subset of the cell_data_set was created in order to include only selected tumors (Table 3). Further comparisons depended on methodology branch (Fig. 10). For example, if the research focused on identifying unique AP-2α/γ target genes within a specific tumor, the remaining cohorts were temporarily excluded from the subset. The study was carried out in accordance with relevant guidelines/regulations.

**Table 3:** Cohorts selected from previous study.

Cohort	Description
BLCA	Bladder urothelial carcinoma
CESC	Cervical and endocervical cancers
COAD	Colon adenocarcinoma
ESCA	Esophageal carcinoma
HNSC	Head and neck squamous cell carcinoma
LUAD	Lung adenocarcinoma
LUSC	Lung squamous cell carcinoma
PAAD	Pancreatic adenocarcinoma
READ	Rectum adenocarcinoma
STAD	Stomach adenocarcinoma
UCEC	Uterine corpus endometrial carcinoma

The plot_cells_3d() was used to visualize the differences between tumors; this relied on the learn_graph() function (with use_partition parameter set as “TRUE”) that was preceded by the reduce_dimension() function (with umap.metric set as “cosine”) and cluster_cells(), both with the reduction method set on UMAP. Finally, the most specific genes (specificity > 0.6) in comparisons were listed using top_markers() with fraction_expressing ≥ 0.5 and marker_test_p_value < 0.05. For tumor vs tumor comparisons, gene expression was plotted on heatmaps generated with the use of the DoHeatmap() function (scale_fill_viridis option “E” was used for aesthetics) after CreateSeuratObject() was used within the Comprehensive R Archive Network (CRAN) Seurat R-package.

### Analysis of correlation, prognostic importance (with validation) and classification model

Genes that met the requirement of specificity > 0.6 were individually correlated with gene encoding AP-2α (*TFAP2A*), AP-2γ (*TFAP2C*) or both (depending on whether the gene was unique or mutual AP-2 target). Correlation AnalyzeR (gccri.bishop-lab.uthscsa.edu/correlation-analyzer) was used to correlate genes using Pearson’s correlation coefficient in desired tissue and sample type (“Gene vs gene” mode was used). Since this tool uses ARCHS4 repository as RNA-seq data source, this could advantageously show relationships independent of TCGA. Survival analysis performed in GEPIA2 (gepia2.cancer-pku.cn) was validated using separate web tools i.e. either pan-cancer RNA-seq KM plotter (kmplot.com) or LOGpc (bioinfo.henu.edu.cn/DatabaseList.jsp), depending on the data availability for specific cancer (e.g. for prognostic endpoints, disease-free or recurrence-free survival (DFS; RFS) was primarily used, with a few examples of disease-specific survival (DSS) and single overall survival (OS)). ROC curves were constructed to evaluate the genes not excluded in previous steps of analysis. Estimation of AUC and 95% Confidence Interval (CI) was done using pROC package with curve visualization made with ggroc and ggplot2 in R environment.

### Evaluation of both immunostaining data and influence on tumor progression

Representative IHC data were obtained from publicly-available Human Protein Atlas (proteinatlas.org); the same antibody for both normal and tumor specimens was selected. The “Tissue” or “Pathology” atlas was used for normal or tumor tissue data, respectively. Genes with prognostic significance were also analyzed on beanplots generated via the BoxPlotR (shiny.chemgrid.org/boxplotr), a web-tool which uses beanplot R-package. The median expression was used as a cut-off value for the post-ROC genes to identify differences in the expression of other genes representing a progression-related signature in a specific tumor. In most cases, two separate scales were used on the Y axis due to large differences in expression level.

## Supplementary Information

Supplementary Information 1.

Supplementary Information 2.

Supplementary Information 3.

Supplementary Information 4.

## Data Availability

The datasets supporting the conclusions of this article are available in the GDAC Firehose repository (https://gdac.broadinstitute.org/) and GitHub (https://github.com/koldam/AP2-prognostic-significance).
