# Supplementary material for: Prognostic significance of AP-2α/γ targets as cancer therapeutics
Source: Sci Rep. 2022 Mar 31;12:5497. doi: 10.1038/s41598-022-09494-1 (PMC8971500; doi:10.1038/s41598-022-09494-1)
Supplement: Supplementary file 1 — Supplementary Information 1. [file 41598_2022_9494_MOESM1_ESM.docx]

**Supplementary Information**

**Supplementary File S1** – 3D trajectory of tumors through AP-2α target genes (html file produced with Plotly).

**Supplementary File S2** – 3D trajectory of tumors through AP-2γ target genes (html file produced with Plotly).

**Supplementary File S3** – AP-2α and AP-2γ target genes from GTRD, TRANSFAC and TRRUST without duplicates (Excel spreadsheets in .xlsx file).
